# Biotic resistance and vegetative propagule pressure co-regulate the invasion success of a marine clonal macrophyte

**DOI:** 10.1038/s41598-018-35015-0

**Published:** 2018-11-09

**Authors:** Elena Balestri, Flavia Vallerini, Virginia Menicagli, Sara Barnaba, Claudio Lardicci

**Affiliations:** Department of Biology, Pisa University, via Derna 1, 56100 Pisa, Italy

## Abstract

Propagule pressure is considered a major driver of plant invasion success. Great propagule pressure would enable invasive species to colonize new areas overcoming the resistance of native species. Many highly invasive aquatic macrophytes regenerate from vegetative propagules, but few studies have experimentally investigated the importance of propagule pressure and biotic resistance, and their interaction, in determining invasion success. By manipulating both recipient habitat and the input of vegetative propagules of the invasive seaweed C*aulerpa cylindracea* in mesocosm, we examined whether higher propagule pressure would overcome the resistance of a native congeneric (*Caulerpa prolifera*) and influence its performance. With the native, *C*. *cylindracea* population frond number decreased irrespectively of pressure level. High propagule pressure did not increase stolon length and single plant size decreased due to the effects of intra- and interspecific competition. Native biomass decreased with increasing *C*. *cylindracea* propagule pressure. These results indicate that higher propagule pressure may fail in enhancing *C*. *cylindracea* invasion success in habitats colonized by the native species, and they suggest that biotic resistance and propagule pressure co-regulate the invasion process. These findings emphasize the need to preserve/restore native seaweed populations and may help to design effective management actions to prevent further *C*. *cylindracea* spread.

## Introduction

The invasion of environments by non-native plant species (NNS) is an increasing threat to biodiversity and functioning of ecosystems globally^1,2^. Numerous factors may individually affect the establishment of a NNS outside its native range^3,4^. Understanding how these factors interact each other to determine the outcome of the invasion process is one of the main goals of ecology, and it is also highly relevant to improving our ability to manage invasive species.

The number of propagules (e.g., individuals, seeds, plant fragments) of a NNS entering the new environment, also termed propagule pressure, is considered a major factor of plant invasion success in a wide range of ecosystems^5–7^. A large propagule input theoretically would enhance the probability of successful invasion enabling an invader to overcome difficulties related to demographic, environmental or genetic stochasticity of a new location^4,8–10^. Relatively few experimental studies, however, have investigated the relationship between propagule pressure and invasion success, and for many plant species it is still unclear whether they may actually gain increasing benefits from increasing propagule pressure^6,11,12^. Moreover, the importance of the interaction between propagule pressure and local factors that can affect plant invasion^7,11,13–15^ has been rarely tested, especially in aquatic environments^16–20^, probably because of the difficulty in measuring and directly controlling the number of propagules introduced in these environments.

Evidence from studies conducted predominantly in terrestrial environments suggests that the nature of interactions between newly arriving species and those already established may play an important role in controlling plant invasion^13,21^. For example, the presence of native species capable of exploiting available key resources (such as light, nutrients and space) may inhibit the establishment or limit the spread of an invader once it has established^21–23^ (biotic resistance hypothesis). Native congeneric species are expected to be more resistant to NNS invasion based on the conjecture that closely related species have similar resource requirements^24^. However, once the invader has established, the impact on the native congeneric may be strong foraging on the same resources. On the other hand, increasing number of studies indicates that in stressful habitats native species may facilitate, for example by physically trapping propagules within the leaf canopy, stabilizing substrates or ameliorating abiotic stresses, rather than compete with NNS^23,25^, which is in direct opposition of predictions of biotic resistance^26^ (facilitation hypothesis). Therefore, in environments where the resistance of resident species is strong, a much higher input of propagules should be necessary to a NNS to maximize its colonization success^27,28^. Instead, in environments where the resistance of resident species is weak, or interactions between resident and NNS are facilitative, only a few propagules should be sufficient to a non-native species to ensure successful invasion^11^.

The invasion success of many highly invasive macrophytes inhabiting aquatic environments is related to the ability to regenerate from detached plant fragments (vegetative propagules) that are passively transported to new locations, in addition to reproduce sexually^29,30^. Among aquatic macrophytes with this feature there are some marine clonal macroalgae^31,32^ (seaweeds), but for most of these species the role of vegetative propagule pressure in determining the colonization of novel habitats remains to be elucidated. The invasion of marine habitats by non-native seaweeds can cause ecological perturbations in local communities and loss of biodiversity with consequent negative impact on ecosystem composition, functioning and services to human society^33^. Understanding the role of vegetative propagule pressure and biotic resistance is crucial not only to improve our knowledge on invasion processes but also to formulate effective strategies to reduce the risk of new marine habitat invasions and the chance of spread of already introduced species, i.e., expend more efforts in restoring and maintaining resident native vegetation if invasion resistance is more important or limiting the release of more propagules if propagule pressure is determinant.

Here, we assessed in mesocosm the individual and combined effects of vegetative propagule pressure (number of propagules per introduction event) and the resistance of a native resident species on the invasion success of a non-native clonal seaweed. In addition, in a separate mesocosm experiment we examined the impact of different levels of propagule pressure (no pressure, low and high pressure) of the invasive species on the performance of the native species. As our non-native species model, we chose *Caulerpa cylindracea* Sonder, since it is a well cited example of successful invasive clonal seaweed. As model native, we used a congeneric clonal species, *Caulerpa prolifera* Forssk J.V. Lamouroux. *Caulerpa cylindracea* is a green seaweed native of Australia that has rapidly spread in the Mediterranean basin during the last decades by invading a variety of habitats, including soft-bottoms and areas formerly occupied by seagrasses, with negative consequences on native primary producers^34–36^. The ability of drifting *C*. *cylindracea* vegetative propagules to establish and grow in nature has been documented^37^, but quantitative data on propagule pressure are not available. Field observations and experimental studies suggest that canopy-forming algal species may either inhibit or facilitate, depending on the species, the invasion of *C*. *cylindracea*, while turfs-forming algae may generally facilitate its spread^38–40^. Disturbances may also promote the spread of the species providing new substrate for colonization while the effect of propagule input seems to have only weak effects on the cover of *C*. *cylindracea*^20,40^. *C*. *prolifera* is a canopy-forming alga that can co-occur with *C*. *cylindracea*. This species is considered not invasive in Mediterranean assemblages, but observations have shown that it had replaced co-occurring native seagrasses in degraded environments^41^. Since *C*. *cylindracea* and *C*. *prolifera* are similar, both functionally and architecturally, we assumed they may compete in situations where resources are limited.

Specifically, we tested the following hypotheses: (1) the establishment of the native species *C*. *prolifera* in the recipient habitat would reduce the colonization success of *C*. *cylindracea* (biotic resistance hypothesis), (2) higher propagule pressure would be necessary to *C*. *cylindracea* to successfully invade a habitat colonized by *C*. *prolifera* (i.e. the effect of propagule pressure on invasion success would be modulated by biotic resistance), and (3) the establishment of *C*. *cylindracea* would reduce the performance of *C*. *prolifera* and this effect would be greater at higher propagule pressure.

## Results

**Figure 1:**
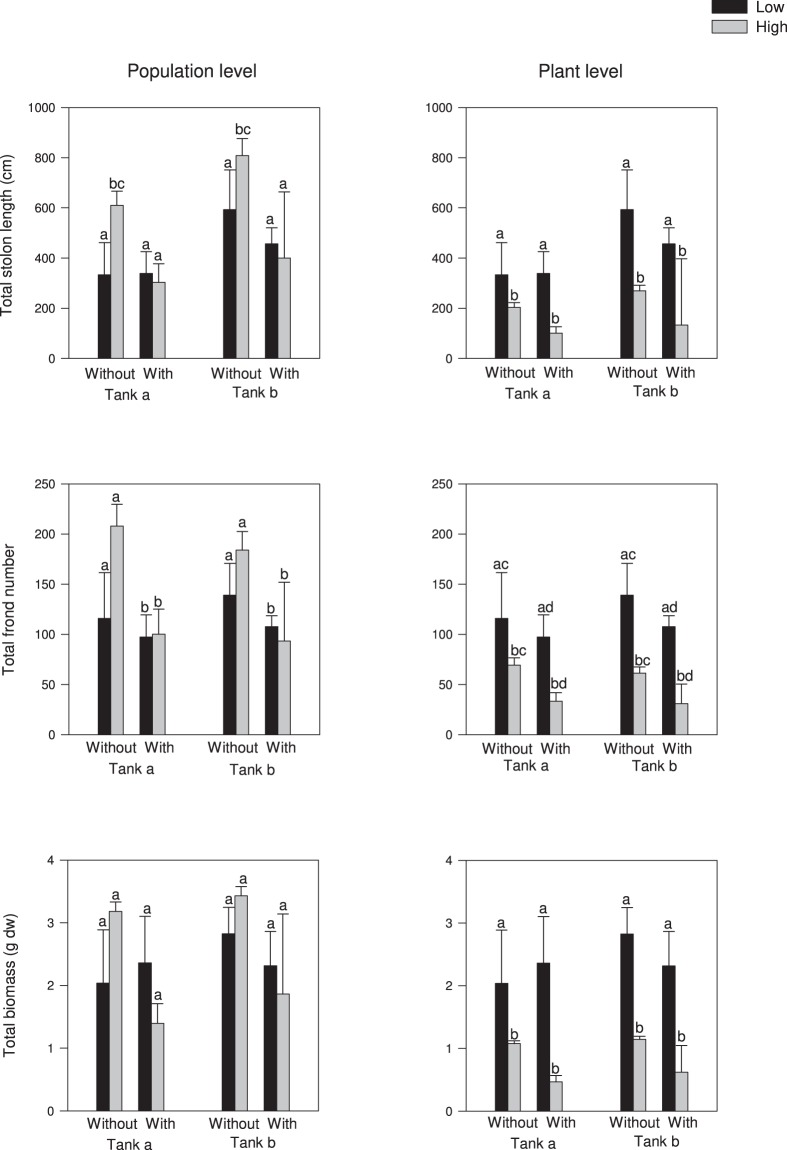
Total stolon length, number of fronds and biomass measured at population level (left panels) and single plant level (right panels) of the invader *C*. *cylindracea* grown with low propagule pressure (Low, one vegetative propagule) and high propagule pressure (High, three vegetative propagules), without (bare sand) and with the native congeneric *C*. *prolifera*, in each of the two tanks (**a**,**b**). Data are means ± SE (n = 3). Letters above bars reports the results of SNK test comparing treatments. Different letters indicate significant differences (P < 0.05).

Two months after experimental invasion, there was at least one established *C*. *cylindracea* propagule in all the mesocosms, thus the greatest probability of invasion was reached irrespective of treatments in both the experiments. At the end of the first experiment, the majority of the established propagules colonized the mesocosm. At population level, total stolon length was substantially affected by the interaction between propagule pressure and native presence treatment (Table 1, Fig. 1). In the absence of *C*. *prolifera*, stolons grown with high propagule pressure were longer than those with low propagule pressure. With high pressure, stolons grown with *C*. *prolifera* were longer than those without *C*. *prolifera*, while no difference was found with low propagule pressure between native treatments. The number of fronds was considerably higher in populations developed without the native irrespective of propagule supply. Instead, total biomass did not differ among treatments. At individual plant level, stolon length and biomass were considerably reduced with high propagule pressure regardless of the native presence/absence (Table 1, Fig. 1). The number of fronds was also reduced with high pressure, and on average there was a higher number of fronds per plant in the absence of the native species than in its presence. No substantial effect from the tank or its interaction with the other factors for all the investigated variables was detected (Tables 1 and 2).

**Table 1:** Summary of 3-way ANOVAs for the effects of vegetative propagule pressure, native species (*C*. *prolifera*) and tank position on growth variables of the invader *C*. *cylindracea* at the level of population and individual plant.

Source	df	Stolon length (cm)		Frond number		Biomass (g dw)	
		F	P	F	P	F	P
Population							
Tank = T	1	3.33	0.0868	0.00	0.9802	0.60	0.4505
Pressure = P	1	23.96	0.1283	3.82	0.3012	139.46	0.0538
Native = N	1	11.97	0.1791	3284.17	**0**.**0111**	33.16	0.1095
T × P	1	0.05	0.8274	0.48	0.4962	0.00	0.9883
T × N	1	0.44	0.5162	0.00	0.9632	0.11	0.7482
N × P	1	219.27	**0**.**0429**	25.00	0.1257	9.14	0.2034
T × P × N	1	0.01	0.9159	0.10	0.7523	0.31	0.5843
Residual	16						
SNK test		No N: High > Low N: High = Low Low: No N = N High: No N > N		No N > N			
Individual plant							
Tank = T	1	3.59	0.0753	0.13	0.7235	0.50	0.4888
Pressure = P	1	16.37	**0**.**0008**	17.14	**0**.**0007**	20.98	**0**.**0003**
Native = N	1	4.38	0.2837	275.14	**0**.**0383**	3.12	0.3279
T × P	1	*		*		*	
T × N	1	0.49	0.4919	0.01	0.9141	0.30	0.5919
N × P	1	1.01	0.4991	0.77	0.5410	1.07	0.4890
T × P × N	1	0.18	0.6726	0.08	0.7778	0.46	0.5086
Residual	16						
SNK test		Low > High		No N > N Low > High			Low > High

Significant results are in bold. *Denotes post-hoc pooling; new F-values are given for those tested against the pooled term. Results of SNK test are reported. No N = absence of the native congeneric *C*. *prolifera* or bare sand, N = presence of the native congeneric *C*. *prolifera*.

**Table 2:** Summary of 2-way ANOVAs for the effect of vegetative propagule pressure on RII values for interspecific interaction and the presence of the native *C*. *prolifera* on RII values for intraspecific interaction based on stolon length, biomass and number of fronds of the invader *C*. *cylindracea*.

Source	df	RII Stolon length		RII Biomass		RII Number of fronds	
		F	P	F	P	F	P
Interspecific interactions							
Tank = T	1	0.61	0.4564	0.49	0.5037	0.22	0.6552
Pressure = P	1	378.94	**0**.**0327**	14.92	0.1613	2970.25	**0**.**0117**
T × P	1	0.01	0.9236	0.27	0.6185	0.001	0.9776
Residual	8						
SNK test		Low < High				Low < High	
Intraspecific interactions							
Tank = T	1	0.58	0.4665	0.41	0.5416	0.87	0.3787
Native = N	1	46.82	0.0924	7.49	0.2230	51.11	0.0885
T × N	1	0.06	0.8083	0.40	0.5459	0.04	0.8490
Residual	8						

Significant results are in bold. Results of SNK test are reported.

**Figure 2:**
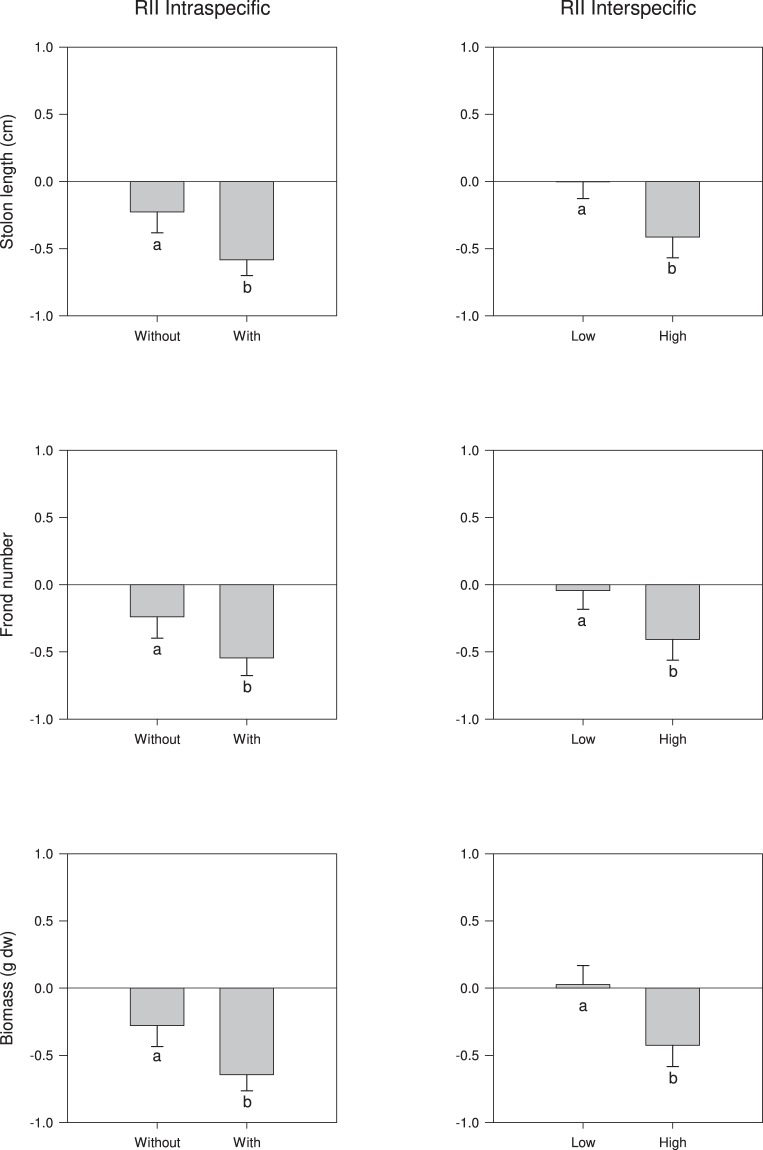
Relative index of intraspecific interaction (RII) based on total stolon length, number of fronds and biomass for *C*. *cylindracea* plants grown with the native and without the native *C*. *prolifera* (left panels), and RII of interspecific interaction calculated on total stolon length, number of fronds and biomass for *C*. *cylindracea* plants grown with low pressure (one vegetative propagule) and high pressure (three vegetative propagules) (right panels). Data are means ± SE (n = 6 as tanks were pooled). Letters above bars reports the outcome of SNK test comparing treatments.

**Figure 3:**
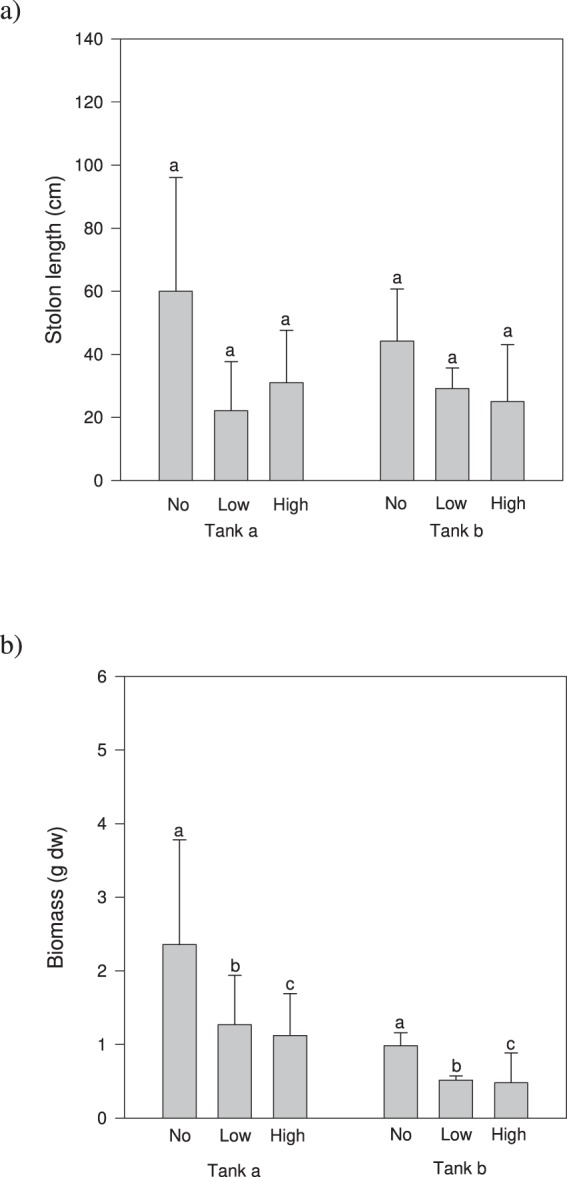
Total stolon length (**a**) and biomass (**b**) of plants of the native congeneric *C*. *prolifera* grown alone (no vegetative propagule of *C*. *cylindracea* or control), with one vegetative propagule (low pressure) and three vegetative propagules (high pressure) of *C*. *cylindracea*, in each of the two tanks (**a**,**b**). Data are means ± SE (n = 3). Letters above bars reports the outcome of SNK test comparing propagule pressure treatments. Different letters indicate significant differences (P < 0.05).

ANOVA did not detect a considerable effect of native treatment on the RII for intraspecific interactions based on biomass, number of fronds and stolon length (Table 2). All the RII values for intraspecific interaction (Fig. 2) were negative but only those relative to plants grown in the presence of *C*. *prolifera* were not statistically different from zero (Table 3). Propagule pressure affected the RII for interspecific interaction on stolon length and number of fronds but did not influence total biomass (Fig. 2, Table 2). All the RIII values for interspecific interactions relative to plants grown with low propagule pressure were not statistically distinguishable from zero while those with high propagule pressure were negative and substantially different from zero (Table 3). In the second experiment, ANOVA did not detect a considerable effect of propagule pressure on total length of stolon of *C*. *prolifera* (Table 4, Fig. 3). Instead, the total biomass of plants grown with low and high propagule pressure was substantially reduced compared to that of control (by about 50% and 65% by respectively, Fig. 3).

**Table 3:** Summary of one-sample mean t test for significant departures from zero (neutral interaction) of RII values based on stolon length, biomass and number of fronds per plant produced by the invader *C*. *cylindracea*, calculated for interspecific interactions at low and high propagule pressures and intraspecific interactions in the presence and absence of the native *C*. *prolifera*. Data from tanks were pooled. Significant results are in bold.

Source	df	RII stolon length		RII biomass		RII number of fronds	
		t	P	t	P	t	P
Interspecific interactions							
Low pressure	5	−0.029	0.9775	0.189	0.8569	−0.311	0.7676
High pressure	5	−269.3	**0.0431**	−269.6	**0**.**0429**	−261.4	**0**.**0473**
Intraspecific interactions							
Without *C*. *prolifera*	5	−145.8	0.2046	−178	0.1351	−152.3	0.188
With *C*. *prolifera*	5	−501.7	**0.004**	−529.6	**0**.**0032**	−415.6	**0**.**0088**

**Table 4:** Summary of 2-way ANOVA on the impact of different levels of vegetative propagule pressure (no pressure, low and high pressure) of the invader *C*. *cylindracea* on the total stolon length and biomass of the native species *C*. *prolifera*. Significant results are in bold. Results of SNK tests are reported.

Source	df	Stolon length		Biomass	
		F	P	F	P
Tank = T	1	0.00	0.9703	2.57	0.1351
Pressure = P	2	15.27	0.0615	220.98	**0**.**0045**
T × P	2	0.08	0.9241	0.00	0.9951
Residual	12				
SNK test				No > Low > High

## Discussion

Our study focuses on still poorly investigated aspects of the invasion of marine environments by non-native clonal macrophytes, i.e., the role of vegetative propagule pressure and its interaction with biotic resistance in determining the outcome of invasion, and the impact of vegetative propagule pressure on the performance of resident species. Ecologists have manipulated propagule supply (i.e. seeds and vegetative propagule) both in the field and in mesocosm to study the invasibility of terrestrial plant communities for decades^42^. To our knowledge, our study is the first attempt to evaluate the effects of vegetative propagule pressure (both at population level and single plant) on the growth and intensity of plant-plant interactions of an invasive seaweed in mesocosm. This experimental approach was chosen as it allows to control not only propagule number and size, but also receiving habitat conditions, minimizing the effects of potential confounding factors such differences in propagule supply and microhabitat characteristics that make it difficult to evaluate the effective role of propagule pressure in invasion success in natural marine habitats.

Our results demonstrate that neither vegetative propagule pressure nor the presence of the native congeneric *C*. *prolifera* in the receiving habitat may influence the establishment probability of the non-native clonal seaweed *C*. *cylindracea*. Indeed, even only a single propagule of *C*. *cylindracea* may successfully establish a new patch irrespective of the absence/presence of the native.

Our first hypothesis (biotic resistance hypothesis) was not fully supported. The presence of *C*. *prolifera* reduced the horizontal spread of *C*. *cylindracea* population only with high propagule pressure. RII for interspecific interaction obtained for all plant growth variables of *C*. *cylindracea* grown in the presence of *C*. *prolifera* and with high propagule pressure indicated a significant competitive effect of *C*. *prolifera* on *C*. *cylindracea*.

Our second hypothesis (higher propagule is necessary to *C*. *cylindracea* to successfully invade a habitat colonized by *C*. *prolifera*) was not supported. With high propagule pressure and in the absence of the native, the total extension of *C*. *cylindracea* populations increased, attaining surprisingly high values (up to 178 m of stolon and 4400 fronds m^−2^), but at the expense of a reduced growth of individual plants. Under this condition, there was only intraspecific interaction, and the slightly negative values of RII for the interaction on all plant growth variables indicated negligible competition between conspecifics. In contrast, in the presence of the native the spread of *C*. *cylindracea* populations did not increase, and the highly negative values of RII for intraspecific interaction on all plant growth variables indicated that the outcome of interaction between conspecifics shifted from neutral to intense competition. However, these latter RII values are based on the response of the invader to conspecifics but also incorporated the effect of the native. Therefore, the jointly effects of intraspecific competition and interspecific competition due to resource (i.e., space and nutrients) limitation possibly cancelled out the positive effects of increased propagule pressure on *C*. *cylindracea* spread. These findings are in contrast with results of previous studies conducted on other invasive clonal macrophytes, which have found that with increased propagule pressure the effect of intraspecific interaction shifted from competition in open habitats to facilitation in vegetated habitats^18,19^. Instead, they are in agreement with studies on some non-clonal species showing that increased propagule pressure may not overcome communities with high resistance^15^. Our results are also in agreement with the lack of consistent effects of high propagule pressure on the growth of *C*. *cylindracea* observed in patches of the seagrass *Posidonia oceanica* L. Delile using higher numbers (5 and 10) of planted manipulated algal fragments^20^.

Our third hypothesis (the establishment of *C*. *cylindracea* reduces the performance of *C*. *prolifera*) was supported. The establishment of even only a single propagule of *C*. *cylindracea* had a considerable impact on biomass production of the native *C*. *prolifera*, resulting in a consistent reduction of biomass (up to 50%) compared to control, and the impact increased with increasing propagule pressure. Although our study was not designed to compare the competitive ability of the two species, the greater biomass reduction observed in a single *C*. *prolifera* individual compared to that observed in a *C*. *cylindracea* one when grown in the presence of one individual of the other species, suggests that this latter species could have higher competitive superiority on the congeneric. This is consistent with a previous study suggesting that *C*. *cylindracea* would be the favoured species in the outcome of the competition with another congeneric, *Caulerpa taxifolia* (Vahl) C. Agardh, invasive in the Mediterranean Sea^43^.

According to some researchers, the competitive superiority of NNS on native ones may be caused by fast growth and maintenance of high density^44^. Here, we found that one *C*. *cylindracea* propagule elongated effectively faster when grown in isolation, producing stolon up to six times longer than one *C*. *prolifera* plant grown alone, despite similar initial fragment length. However, when one *C*. *cylindracea* propagule was grown with one *C*. *prolifera* plant the number of fronds significantly decreased and the plant assumed a more “guerrilla” growth form i.e., spreading ramets formed a loosely arranged group of widely spaced ramets^45^. Modelling studies indicate that plants with a guerrilla growth form are generally specialized in the occupation of free space, and the long spacers between ramets allow them infiltration in the surrounding vegetation as well as escaping from less favorable patches where resource levels are low or competitive stress is high^46,47^. Therefore, faster elongation and capacity to adjust clonal growth form could provide to *C*. *cylindracea* an advantage over the congeneric under low propagule pressure, enabling it to more efficiently monopolize available space. Instead, with higher propagule pressure increased inter- and intraspecific competition could limit the capacity of *C*. *cylindracea* to adjust its growth form to escape from *C*. *prolifera*. Clearly further studies are needed to examine more in detail the shape of the relationship between propagule pressure and colonization success in the field and across a range of propagule supply and habitat conditions.

Overall, the results of the present study provide new experimental evidence of the high invasion potential and spread of *C*. *cylindracea*. They also demonstrate that biotic resistance and propagule pressure may co-regulate the invasion process of this species, and that vegetative propagule pressure is not always a good predictor of invasion success. In addition, the results suggest that in nature a relatively high input of propagules (three) of *C*. *cylindracea* may favour the colonization of bare substrate patches but the same propagule input may not be sufficient to maximize the invasion success of areas colonized by a native competitor. However, the loss of native vegetation due to disturbances by releasing plants from interspecific competition could locally promote the spread of *C*. *cylindracea*, in accordance with the large invasion of disturbed areas described in most previous field studies^20,40^. Our findings may have relevant ecological and management implications. Firstly, in most previous field and observational studies comparing the invasibility of different habitats by clonal seaweeds, the interaction between vegetative propagule pressure and biotic resistance has formally not been investigated. Failure to include this factor can make it difficult to determine if differences in invasibility across habitats are effectively due to differential susceptibility to invasion (or resistance of resident communities) or rather result from variations in propagule input^48,49^. Even when the invasion extent of two different habitats is similar, the mechanisms of invasion may differ; for example, in one habitat (as observed, for example in a bare substrate) the invader might have established under low propagule pressure while in the other one (as found here, for example in a vegetated substrate) the invader might have established under high propagule pressure.

From a management point of view, the approach required to prevent or control seaweed invasion would differ according to the nature and intensity of interaction between the invasive and the native species in the recipient habitat. For example, in habitats where the effects of intra- and interspecific competition are important as observed here in the presence of the congener *C*. *prolifera*, high propagule pressure may contribute little to enhance invasion success. Under these conditions, strategies such as minimizing propagule release or eradicating already established propagules of the invasive species could not provide benefits, and managers should thus expend efforts on conserving existing algal communities and restoring degraded ones to reduce the chance of establishment and growth. On the other hand, in habitats where propagule pressure may be important, as our results suggest for open/disturbed habitats, managers should focus on minimizing the risk of new propagule introductions to limit the spread of the invasive species.

## Materials and Methods

### Experimental design

We conducted two separate mesocosm experiments in an outdoor aquaculture system set up on a coastal dune area at the INVE Aquaculture Research Center of Rosignano Solvay (Italy), and equipped following a protocol established for successfully growing marine plants^50^. The first experiment examined the main and interactive effects of propagule pressure and native species presence on the colonization success (establishment and spread) of the non-native invasive *C*. *cylindracea*. The second experiment assessed the impact of different levels of propagule pressure of *C*. *cylindracea* on the clonal growth of the native.

Before the start of the experiments (March 2015), plastic growth containers (240 mm diameter × 220 mm height), filled with natural sediment (carbonate sand) collected at 0.5 m depth near the Aquaculture Centre (North Western Mediterranean Sea), were equally distributed in two tanks (10000 L) full of natural seawater. The sediment was previously carefully mixed and sieved (0.2 mm). During the experimental period, continuous seawater flux maintained a constant level of water (1.5 m) above the containers (thereafter referred as mesocosms) in each of the two tanks. Seawater temperature ranged from 16 to 27.8 °C, pH was 8.2 and salinity varied between 38 and 38.2 throughout the experiments. In April 2015, vegetative propagules of *C*. *cylindracea* were collected along the shoreline after a storm while *C*. *prolifera* fragments were collected from a bed near to the Aquaculture Center. All plant material was transported in seawater from the sampling site to the laboratory for plant measurements (stolon length and number of erect fronds). To standardize propagule size, we selected propagules of *C*. *cylindracea* of similar size (stolons 10 cm ± 1 cm SE long, each with 2–3 erect fronds). Fragments of *C*. *prolifera* were gently washed free of any adhering sediment particle and manually cut into fragments of equal size to that of selected *C*. *cylindracea* propagules. A sample of selected propagules and fragments (n = 10) was dried at 60 °C to constant mass and weighed to determine initial plant biomass (0.062 ± 0.012 g dry weight (dw) for *C*. *prolifera* and 0.041 ± g dw for *C*. *cylindracea*) while the remaining ones were left to acclimate to tank conditions for three days before their use.

For the first experiment, six mesocosms were planted with a single stolon fragment of *C*. *prolifera* and six mesocosms were left unplanted in each of two tanks. After *C*. *prolifera* establishment (3 days), each mesocosm was artificially invaded by adding one propagule (low pressure, corresponding to approximately 25 propagules m^−2^) or three propagules (high pressure, corresponding to about 75 propagules m^−2^) of *C*. *cylindracea*. There were therefore four treatments: one propagule or three propagules of *C*. *cylindracea* in bare habitat thus in the absence of interspecific interaction, and one propagule or three propagules of *C*. *cylindracea* planted with the native species *C*. *prolifera* thus in the presence of interspecific interaction. There were three replicates per treatment in each of the two tanks, in total 24 mesocosms.

For the second experiment, nine mesocosms were planted with a single fragment of *C*. *prolifera* in each of two tanks. After *C*. *prolifera* established, the mesocosms were randomly attributed to one of the following treatments: no exposure to the invader *C*. *cylindracea* (one *C*. *prolifera* individual alone or control), low pressure (one *C*. *prolifera* individual subjected to the invasion by one propagule of *C*. *cylindracea* with) or high pressure (one *C*. *prolifera* individual subjected to the invasion by three propagules of *C*. *cylindracea)*. There were three replicates per treatment in each of the two tanks, 18 mesocosms in total.

In both the experiments, *C*. *prolifera* fragments were planted in centre of the mesocosms and *C*. *cylindracea* propagules were placed in randomly chosen positions at the edge of the *C*. *prolifera* fragment. The mesocosms were randomly assigned to new positions in each of the two tanks every month to minimize biases caused by variations across the different areas within the tank. The experiments lasted for six months to cover the whole growing season of *C*. *cylindracea* in the Mediterranean Sea^51^. After this period, all plants were carefully harvested, washed with seawater to remove sand and transported to the laboratory for clonal growth measurements (total length of stolons and number of erect fronds). All the plants were then dried to constant mass at 60 °C and weighed to determine total biomass. Growth measures as well as total biomass were calculated both at population level (mesocosm) and individual level (single plant).

### Statistical analysis

In both the experiments, the number of propagules of *C*. *cylindracea* established (rooted in the substrate) in each mesocosm was recorded twice per week for the first two months to estimate the probability of successful invasion, expressed as proportion of mesocosms in each treatment with one or more established clones of *C*. *cylindracea* at the end of the first two months (Fig. 3). Given we had three replicates per treatment in each tank, the probability of success was constrained to four possible values (0, 0.33, 0.66, or 1.0).

In the first experiment, three-way analyses of variance (ANOVAs) with categorical predictor variables for fixed effects of native congeneric (two levels, absent and present) and propagule pressure (two levels, one and three propagules), and random effect of tank position (two levels, a and b) were separately performed for each selected response variable measured at both individual level and population level to test for the main and interactive effects of propagule pressure and native species presence.

Separate two-way ANOVAs were used to assess the effect of native species and tank position on the index of relative intensity of intraspecific interaction, RII^52^, and the effect of propagule pressure and tank position on the RII for interspecific interaction based on stolon length, number of fronds and biomass data of *C*. *cylindracea*. The intraspecific RII was calculated as follows:

$${\rm{Intraspecific}}\,{\rm{RII}}=({{\rm{Y}}}_{{\rm{high}}}-{{\rm{Y}}}_{{\rm{low}}})/({{\rm{Y}}}_{{\rm{high}}}+{{\rm{Y}}}_{{\rm{low}}})$$

where Y_high_ is the mean growth measure of a *C*. *cylindracea* propagule grown with high propagule pressure and Y_low_ is that measure with low propagule pressure. The index was calculated for each of the two conditions, *C*. *prolifera* absent and present. The interspecific RII was calculated as follows:

$${\rm{Interspecific}}\,{\rm{RII}}=({{\rm{Y}}}_{{\rm{with}}}-{{\rm{Y}}}_{{\rm{without}}})/({{\rm{Y}}}_{{\rm{with}}}+{{\rm{Y}}}_{{\rm{without}}})$$

where Y_with_ is the mean growth measure of a *C*. *cylindracea* propagule with *C*. *prolifera* and Y_without_ is that measure without *C*. *prolifera*. The index was calculated for each of the two propagule pressure conditions, low and high propagule pressure. RII has defined limits [−1, 1], and is negative when competition prevails, positive when there is prevalence of facilitation and zero when there is no interaction. For each of the calculated RII values significant departures of mean RII values from zero were assessed by using one-sample mean t-test (two-tailed t test, StatSoft version 6.0^53^) on data tanks pooled.

In the second experiment, two-way ANOVAs with categorical predictor variables for fixed effects of propagule pressure (control, one propagule or three propagules of *C*. *cylindracea*) and random effect of tank position (a and b) were performed separately for total stolon length and biomass of *C*. *prolifera* to evaluate the impact of propagule invasion on the native performance.

Prior to all analyses, data were assessed for normality and homogeneity of variance using Shapiro-Wilk test and Cochran’s C tests (α = 0.05), respectively. In the second experiment, data on the stolon length and biomas*s* of *C*. *prolifera* were ln (x + 1) transformed to meet the assumption of homogeneity of variance and normality. When significant main effects were found in the ANOVAs, the means were compared using the Student-Newman-Keuls (SNK test, at α = 0.05) test. All ANOVA analyses were conducted with the software—GMAV version 5.0 for Windows^54^.

## Data Availability

The datasets generated and analyzed during the present work are available from the corresponding author on reasonable request.
